# Biobehavioral Interactions Between Stress and Alcohol

**DOI:** 10.35946/arcr.v40.1.04

**Published:** 2019-10-17

**Authors:** Marcus M. Weera, Nicholas W. Gilpin

**Affiliations:** Marcus M. Weera, Ph.D., is a postdoctoral fellow in the Department of Physiology, Louisiana State University Health Sciences Center, New Orleans, Louisiana. Nicholas W. Gilpin, Ph.D., is a professor in the Department of Physiology, Louisiana State University Health Sciences Center, New Orleans, Louisiana

**Keywords:** alcohol, animal models, stress

## Abstract

In this review, the effects of stress on alcohol drinking are discussed. The interactions between biological stress systems and alcohol drinking are examined, with a focus on the hypothalamic pituitary adrenal axis, corticotropin releasing factor, dynorphin, neuropeptide Y, and norepinephrine systems. Findings from animal models suggest that these biological stress systems may be useful targets for medications development for alcohol use disorder and co-occurring stress-related disorders in humans.

## Behavioral Interactions Between Stress and Alcohol

Epidemiological studies of humans suggest that stress increases alcohol drinking. For example, findings from the 2001–2002 National Epidemiologic Survey on Alcohol and Related Conditions show that the number of past-year stressors is positively associated with prevalence of current drinking, current binge drinking, and alcohol use disorder (AUD) diagnosis.^1^ However, as with most epidemiological human studies, the temporal and causal relationships between stress exposure and alcohol drinking are difficult to determine. Therefore, studies using animal models represent a useful complement for examining relationships between stress and alcohol drinking. Keyes and colleagues reviewed key epidemiological findings that show that stress exposure is associated with increased risk for AUD.^1^

Historically, studies using animal models to test the relationship between stress and alcohol drinking have focused on stress-induced reinstatement of alcohol-seeking as a model of stress-induced alcohol relapse in humans. In this procedure, animals are trained to self-administer alcohol in an operant task, that behavior is then extinguished (by omitting alcohol as reinforcement for lever pressing), after which exposure to a stressor (e.g., footshock) reinstates lever pressing for alcohol (i.e., alcohol-seeking).^2^ In fact, stress has consistently been shown to reinstate seeking of a variety of drugs, including heroin, cocaine, and nicotine.^3^

A more limited body of literature shows that stress may increase alcohol consumption, but this effect depends heavily on a number of factors, including the stressor and the alcohol-drinking model used, as well as the species, sex, and age of the experimental animals.^4^ Studies that show stress-induced escalation of alcohol drinking in rodents, with or without prior experience of alcohol drinking, are summarized in Table 1.^5^–^11^ Stress also can synergize with exposure to high doses of alcohol to produce faster and more robust escalation of alcohol drinking in mice.^12^ However, it is noteworthy that many stress procedures do not produce escalated alcohol drinking in rodents, and there is a paucity of animal models for studying stress-induced escalation of alcohol drinking and related behaviors (e.g., anxiety).^13^,^14^

On the other hand, chronic exposure to high doses of alcohol (which is an animal model of alcohol dependence) increases stress reactivity during withdrawal. For example, rats^15^ and mice^16^ exposed to chronic high-dose alcohol, followed by restraint stress during withdrawal, show higher levels of stress-induced anxiety-like behavior (in the elevated plus maze test) and suppression of social interaction, respectively, compared to their alcohol-naïve counterparts.

Data from animal models suggest that stress may not only trigger relapse to alcohol drinking but also increase subsequent alcohol drinking. Animal studies also show that exposure to high doses of alcohol increases stress reactivity. These studies suggest that stress exposure may facilitate development of AUD in humans, which may increase the likelihood of developing a stress-related disorder, further exacerbating AUD. The precise mechanisms through which this occurs are unclear, but dysregulation of brain stress signaling systems is likely to play a central role. Stress and chronic alcohol exposure alter the activity of brain stress systems, and dysregulation of these systems has demonstrable effects on alcohol drinking. The next section summarizes key findings from animal studies regarding the interaction between alcohol and brain stress systems.

## Neurobiological Interactions Between Stress and Alcohol

Although alcohol often is consumed to alleviate stress,^1^ alcohol may activate some brain stress systems and may be considered a stressor itself.^17^ A body of literature shows that dysregulation of brain stress systems induced by stress or chronic high-dose alcohol exposure contributes to escalation of alcohol drinking or to alcohol-seeking relapse. This section summarizes key findings from research on several brain stress systems that likely mediate stress-alcohol interactions.

### Hypothalamic pituitary adrenal axis

One of the main physiological responses to stress is activation of the hypothalamic pituitary adrenal (HPA) axis. This process begins with release of corticotropin releasing factor (CRF) from cells in the paraventricular nucleus of the hypothalamus, which leads to increased release of adrenocorticotropic hormone in the pituitary, which stimulates glucocorticoid (cortisol in humans and corticosterone in rodents) release in the adrenal gland. Therefore, HPA activation is generally considered to be “pro-stress,” but the effects of HPA activity and corticosterone level on stress-related outcomes (e.g., anxiety-related behaviors) may depend on several factors. In animals, administration of corticosterone systemically or into the brain increases alcohol drinking,^18^ and systemic glucocorticoid receptor blockade with mifepristone reduces alcohol drinking,^19^ suggesting that glucocorticoid signaling modulates alcohol drinking. In addition, research has shown that infusion of mifepristone into the central amygdala attenuated stress-induced reinstatement of alcohol-seeking,^20^ suggesting that glucocorticoids act on specific brain regions to modulate alcohol relapse-like behavior.

Interestingly, in a study that used a predator odor stress model, a blunted plasma corticosterone response in rats following predator odor exposure predicted high stress reactivity (avoidance of a stress-paired context).^21^ Also, systemic corticosterone treatment before the stress exposure reduced the percentage of animals that were highly stress reactive (Avoiders) and reduced the magnitude of their stress reactivity (avoidance).^22^ After stress, the Avoiders exhibited increased alcohol drinking, as compared to the Non-Avoiders,^10^ which suggests that failure to mount a proper HPA response to traumatic stress predicts later escalation of alcohol drinking, which is similar to the notion that failure to mount a proper HPA response to traumatic stress predicts later post-traumatic stress disorder pathology^23^ and poor treatment response^24^,^25^ in humans.

Studies of rodents have demonstrated that acute alcohol exposure (experimenter-administered or self-administered) stimulates corticosterone release, mimicking a stressor.^26^,^27^ In one study that used a model of chronic, high-dose alcohol exposure, alcohol-dependent rats, when compared with control rats, showed lower basal corticosterone levels during withdrawal and smaller increases in corticosterone following experimenter-administered or self-administered alcohol.^27^ However, this effect may depend on factors such as the rodent species^28^ and whether total or free amounts of glucocorticoids were measured.^29^ This response is akin to the blunted corticosterone response shown in Avoider rats following exposure to traumatic stress.

In addition, a high basal corticosterone level in rats has been shown to protect against stress-induced and corticosterone injection–induced exacerbation of anxiety-like behavior.^30^ Therefore, a blunted corticosterone response to alcohol or stress may be a common mechanism through which chronic, high-dose alcohol or traumatic stress increases alcohol drinking and stress-related disorders. However, Perusini and colleagues found that inhibition of corticosterone synthesis before stress blocked stress-enhanced fear conditioning.^31^

Studies of rats also have shown that glucocorticoid receptor levels in the brain were elevated following chronic alcohol exposure, and that mifepristone blockade of glucocorticoid receptors in these rats, systemically or within the central amygdala, reduced escalation of alcohol drinking.^32^ Collectively, these findings suggest that HPA function and glucocorticoid receptor signaling in the brain, perhaps in specific brain regions, are important targets for medications development for AUD and co-occurring stress-related disorders.

### CRF system

Aside from being a critical component of the neuroendocrine stress response, CRF signaling in extrahypothalamic brain regions is also a critical mediator of stress-alcohol interactions. For example, intraventricular infusions of a CRF receptor antagonist have been shown to attenuate stress-induced reinstatement of alcohol-seeking in rats,^33^ and systemic blockade of CRF_1_ receptors has produced similar effects.^34^ Systemic CRF_1_ receptor blockade also has been shown to reduce escalated alcohol drinking after exposure to stress induced by predator odor (in rats)^35^ or by social defeat (in mice).^36^ In studies of alcohol-dependent animals, intraventricular infusions of the CRF receptor antagonist D-Phe-CRF(12-41) reduced escalated alcohol drinking for both rats^37^ and mice^38^ during withdrawal. This effect is mediated, at least in part, by the central amygdala, as infusion of D-Phe-CRF(12-41) into the central amygdala also has been shown to reduce escalated alcohol drinking in alcohol-dependent rats during withdrawal.^39^ CRF effects on escalated alcohol drinking appear to be mediated largely by the CRF_1_ receptor. For example, researchers have reported that systemic CRF_1_ receptor blockade reduced escalated alcohol drinking in mice^40^ and rats^41^ after chronic exposure to high doses of alcohol.

Collectively, these findings suggest that neural processes mediated by CRF–CRF_1_ receptor signaling play an important role in escalation of alcohol drinking, and in alcohol-seeking relapse, induced by stress or by chronic, high-dose alcohol exposure. For more detailed discussions of this topic, please refer to reviews by Phillips and colleagues,^42^ Spierling and Zorrilla,^43^ and Pomrenze and colleagues.^44^

### Dynorphin system

Stress generally increases brain dynorphin levels,^45^ and dynorphin signaling via kappa-opioid receptors (KORs) mediates stress effects on behavior. For example, chronic stress (repeated forced-swim or repeated footshock stress) has been shown to produce dysphoria-like behaviors in mice that can be attenuated by systemic KOR blockade or by gene deletion.^46^ In one study, systemic administration of KOR antagonists reduced stress-induced escalation of alcohol drinking and alcohol-induced place preference in mice.^47^ In another study, systemic KOR blockade attenuated reinstatement of alcohol-seeking in rats, which had been induced by yohimbine (an alpha_2_-adrenergic receptor antagonist often used as a pharmacological stressor).^48^

These results are complemented by findings that dynorphin-KOR signaling in the brain is enhanced by chronic, high-dose alcohol exposure. For example, alcohol-dependent rats, relative to nondependent controls, have been shown to exhibit higher dynorphin levels and increased KOR function in the amygdala during withdrawal.^49^ In the same study, KOR blockers, administered systemically or directly into the central amygdala, reduced escalated drinking in alcohol-dependent rats during withdrawal. Reviews by Anderson and Becker^50^ and Karkhanis and colleagues^51^ provide further discussion on the role of this system in stress-alcohol interactions.

### Neuropeptide Y system

In contrast to the CRF and dynorphin systems, the neuropeptide Y system is generally thought to produce anti-stress effects. For example, following predator odor exposure, rats that exhibited high stress reactivity had lower neuropeptide Y levels in the brain, relative to rats that had lower stress reactivity.^52^ In the same study, an infusion of neuropeptide Y into the brain an hour after stress exposure reduced the number of rats that subsequently exhibited high stress reactivity. In another study, neuropeptide Y infusion into the brain, followed by yohimbine-induced stress, attenuated reinstatement of alcohol-seeking.^53^

Compared to alcohol-naïve controls, alcohol-dependent rats have been shown to exhibit lower neuropeptide Y expression in several brain areas associated with negative affect and motivation, including amygdalar, cortical, and hypothalamic subregions.^54^ These results suggest that chronic, alcohol-induced neuropeptide Y deficits in the brain may contribute to escalation of alcohol drinking and to negative affect during withdrawal. In other studies, an intracerebroventricular infusion of neuropeptide Y into the whole brain^55^ or specifically into the central amygdala^56^ reduced escalation of alcohol drinking in alcohol-dependent rats, suggesting that modulation of neuropeptide Y signaling in the brain may have therapeutic value in the treatment of AUD.

Both neuropeptide Y receptor subtypes (Y_1_ and Y_2_) have demonstrated roles in regulating alcohol drinking in rodents. For instance, intraventricular infusion of a Y_1_ receptor agonist or a Y_2_ receptor antagonist has been shown to reduce alcohol drinking in mice.^57^ In a study of rats, the ability of a Y_2_ receptor antagonist (via intracerebroventricular administration) to reduce alcohol drinking may have been potentiated in animals that were chronically exposed to high-dose alcohol.^58^ However, Kallupi and colleagues found that a Y_2_ receptor antagonist (administered systemically or into the central amygdala) attenuated only anxiety-like behavior, but not alcohol drinking, in rats chronically exposed to high-dose alcohol.^59^

Researchers have reported that Y_1_ and Y_2_ receptors regulate alcohol drinking in a brain region–specific manner. For example, research has demonstrated that Y_1_ receptor activation or Y_2_ receptor blockade in the medial prefrontal cortex reduced alcohol drinking in mice,^60^ whereas Y_1_ receptor activation in the paraventricular nucleus increased alcohol drinking in rats.^61^ Further discussions of this topic can be found in reviews by Robinson and Thiele^62^ and Thorsell and Mathé.^63^

### Norepinephrine system

The locus coeruleus is densely packed with noradrenergic neurons that project to specific brain nuclei in the amygdala, prefrontal cortex, and hippocampus and that are important in the regulation of emotion and motivation.^64^ Stress engages some of these projections. For example, in a study of rats, immobilization stress increased norepinephrine release in the central amygdala.^65^ In a different study of the central amygdala, alpha_1_-adrenergic receptor blockade with prazosin reduced stress-induced augmentation of anxiety-like behavior.^66^ Research has also demonstrated that prazosin blocked stress-induced reinstatement of alcohol-seeking in rats.^67^ In a study of rats chronically exposed to high-dose alcohol, administration of prazosin^68^ or the beta-adrenergic receptor blocker propranolol^69^ blocked escalation of alcohol drinking during alcohol withdrawal.

Stress and chronic alcohol exposure also increase the activity of the sympathetic nervous system (a subdivision of the autonomic nervous system, which mediates the flight-or-fight response) and thereby affect the function of many organ systems, in part through increased noradrenergic signaling. For example, psychosocial stress in mice has been shown to increase blood pressure via an alpha_1_-adrenergic receptor–dependent mechanism.^70^

During withdrawal from chronic, high-dose alcohol exposure, increases in sympathetic activity contribute to aversive physiological symptoms, such as increased blood pressure, heart rate, and sweating, which are thought to contribute to relapse in abstinent individuals.^71^ In studies of rats, blockade of alpha_1_- and beta-adrenergic receptors^72^,^73^ and activation of alpha_2_-adrenergic autoreceptors^73^ reduced alcohol withdrawal symptoms such as convulsions, tremors, and locomotor hyperactivity. In another study of rats, blockade of norepinephrine signaling during withdrawal attenuated alcohol drinking.^68^ See the review by Vazey and colleagues^74^ for further discussion of this topic.

## Conclusion and Future Directions

Brain stress systems mediate the effects of stress on alcohol drinking and the effects of chronic alcohol exposure on subsequent alcohol drinking and stress reactivity. Therefore, brain stress systems are useful targets for the development of medications for AUD and for co-occurring stress-related disorders. More specifically, glucocorticoid, CRF, dynorphin, neuropeptide Y, and norepinephrine systems may be useful targets for modulating stress-alcohol interactions. Several pharmacological agents that target these systems are promising candidates for the treatment of AUD and co-occurring mental health conditions in humans.^75^ In addition, emerging evidence has shown that several other brain stress signaling systems, such as oxytocin,^76^ nociceptin,^77^,^78^ and neuropeptide S,^79^ also contribute to stress-alcohol interactions, suggesting they also may be promising therapeutic targets. To guide medications development for AUD and co-occurring stress-related disorders, future studies should elucidate the mechanisms through which stress-related neuropeptide and neurotransmitter systems affect alcohol- and stress-related behaviors, including how these systems interact or modulate glutamate and gamma-aminobutyric acid (GABA) neurotransmission in specific circuits.^80^,^81^

## Figures and Tables

**Table 1:** Studies of Stress-Induced Escalation of Alcohol Drinking in Rodents

Procedure	Developmental Stage at Exposure	Stressor	Alcohol-Drinking Procedure
Stress → Alcohol Drinking			
In Rats	Adult	Repeated footshocks^5^	Two-bottle choice drinking
	Adolescent	Postweaning social isolation^6^*	Two-bottle choice drinking and operant self-administration
In Mice	Adult	Repeated social defeat^7^	Two-bottle choice drinking
	Adolescent	Postweaning social isolation^8^	Two-bottle choice drinking
Alcohol Drinking → Stress → Alcohol Drinking			
In Rats	Adult	Single exposure to soiled cat litter^9^†	Two-bottle choice drinking
	Adult	Single exposure to bobcat urine^10^†‡	Operant self-administration
In Mice	Adult	Repeated social defeat or forced swim^11^	Two-bottle choice drinking

* Stress increased alcohol drinking only in male rats.

† Stress increased alcohol drinking only in rats that were highly stress reactive.

‡ Stress increased responding for quinine-adulterated alcohol (aversion-resistant responding) in rats that were highly stress reactive.
